# The potency of bacteriophages isolated from chicken intestine and beef tribe to control biofilm-forming bacteria, *Bacillus subtilis*

**DOI:** 10.1038/s41598-023-35474-0

**Published:** 2023-05-22

**Authors:** Agustin Krisna Wardani, Efendi Oulan Gustav Hakim Nata Buana, Aji Sutrisno

**Affiliations:** Department of Food Science and Biotechnology, Universitas Brawijaya, Malang, 65145 Indonesia

**Keywords:** Biotechnology, Microbiology

## Abstract

Biofilm becomes one of the crucial food safety problems in the food industry as the formation of biofilm can be a source of contamination. To deal with the problem, an industry generally employs physical and chemical methods including sanitizers, disinfectants, and antimicrobials to remove biofilm. However, the use of these methods may bring about new problems, which are bacterial resistance in the biofilm and the risk for product contamination. New strategies to deal with bacterial biofilms are needed. Bacteriophages (phages), as a green alternative to chemical, have re-emerged as a promising approach to treat bacterial biofilm**.** In the present study, the potential of lytic phages which have antibiofilm activity on biofilm-forming bacteria (*Bacillus subtilis*), were isolated from chicken intestines and beef tripe obtained from Indonesian traditional markets using host cells obtained isolated from these samples. Phages isolation was conducted by using double layer agar technique. A lytic test of phages was administered on biofilm-forming bacteria. The difference of turbidity level between control (which were not infected by phages) and the test tubes containing host bacteria infected by phages was investigated. The infection time for the production of phages was determined based on the level of clarity of the media in the test tube with a longer lysate addition time. Three phages were isolated namely: ϕBS6, ϕBS8, and ϕUA7. It showed the ability to inhibit *B. subtilis* as biofilm-forming spoilage bacteria. The best inhibition results were obtained from ϕBS6. Infection with ϕBS6 in *B. subtilis* lead to 0.5 log cycle decreased in bacterial cells. This study showed that isolated phages might be used as a potential approach for handling the problem of biofilm formation by *B. subtilis*.

## Introduction

Recent outbreaks of foodborne illness can be attributed to biofilms. Biofilm formation is an integral part of the microbial life cycle in nature. Foodborne pathogens form biofilms as a survival strategy in various unfavorable environments, which also become a frequent source of recurrent contamination and outbreaks of foodborne illness^1^. Approximately 60 percent of foodborne illness outbreaks are caused by biofilms, according to food safety research^2^. Biofilm is a form of bacterial adaptation that colonizes and attaches to the surface, covered by extracellular polymeric material^3^. It becomes a major problem in the food, health, and marine industries^4^. Eighty percent outbreaks due to pathogenic bacteria are contributed by biofilm-forming bacteria^5^. The formation of biofilms by pathogenic bacteria, especially in the processing equipment, is a big challenge in the food industry. In the food industry—such as the brewing industry, dairy product processing, fresh product, and meat—the presence of biofilm within the production line will be a source of contamination for foodstuffs that pass through the production line^6–8^. The formation of biofilms also generates a negative impact on non-food industry such as the oil drilling industry, paper production, health products/medicines^9,10^. Biofilms bring about difficulties during the production process because they can reduce heat transfer, block tubes, filters, and it causes surface damage to equipment^11,12^.

The strategy that has so far been applied by industries to control the formation of biofilms is using chemicals, such as acid, oxidizing compounds (chlorine, H_2_O_2_), and surfactants^13–15^. However, the use of these chemicals induces limitations. It can pose a risk of cross-contamination and cause poisoning. The main challenge in preventing the formation of biofilms is that they are known to have high resistance to antimicrobials, antibiotics, disinfectants, and sanitizers, making them difficult to remove^16–19^. Besides, biofilms cause bacteria to have a higher resistance to high temperatures, UV rays, x-rays, drought and so on^20–22^. New approach is needed to control biofilms. One of the potential alternative solutions that can be explored is the use of bacteriophages (bacterial viruses) capable of lysing biofilm-forming bacteria^23,24^. *Bacillus spp.* is one of the bacteria that often generates problems related to the formation of biofilms in the food processing industry^25,26^. The biocontrol strategy through bacteriophages to reduce biofilms is an acceptable strategy today because it is more natural than the conventional methods, with chemicals, to improve food safety. Change in trend of consumers to natural from chemical and also the increasing health and environmental concerns creating demand for new "green" technology in food processing. Until now, several phages and endolysins have been used to inhibit the formation of biofilms of several bacteria, which are *E. coli* O157:H7, *Bacillus* spp., *Salmonella* spp., *Campylobacter* spp., *Listeria* spp., *Staphylococcus* spp., *Cronobacter* spp., *Vibrio* spp., *Clostridium* spp., *Campylobacter jejuni* and *Pseudomonas* spp.^27–29^.

The current study aimed to isolate bacteriophages from chicken intestine and beef tripe, which had the ability to inhibit the biofilm-forming bacteria, *Bacillus subtilis*. Evaluation was carried out on the lytic ability of bacteriophages that had been isolated against several target bacteria. From this study we expected that isolated phage can be a candidate as a strategy to combat biofilm formation and as an alternative among conventional methods.

## Material and methods

### Sample preparation

The samples used were chicken intestine and beef tripe obtained from Indonesian traditional markets. They were approximately 5 g of chicken intestine and beef tripe, each of which was put in two separate plastics with 45 mL of distilled water (dilution treatment 10^–1^). The chicken intestine and beef tripe samples were then homogenized at high speed for 60 s. The aliquots of the chicken intestines and beef tripe were ready for the isolation of host bacteria and bacteriophages.

### Bacteriophage isolation

Phage isolation was made based on the method proposed by^30^. A total of 15 mL of aliquot sample was centrifuged at 6500 rpm at a temperature of 27 °C. The supernatant was taken by using a syringe and filtered with 0.22 µm sterile filter. The obtained filtrate was free from bacteria and presumably contained the desired bacteriophage. A total of 100 µL of phage filtrate and 100 µL of 0.3 M CaCl_2_ were added to 3 mL of soft NA containing 100 µL of host bacteria aged 20 h. The mixture in the soft NA was vortexed to mix well. Then, the soft NA was poured on top of the hard NA in the petri dish and allowed to solidify to form a double layer. Double layer agar was incubated at 37 °C for 24 h. Phage will be found when the double layer agar contains plaque. The isolated phages tested in this study were stored at − 4 °C and available in the Biotechnology Laboratory, Universitas Brawijaya, Malang.

### Host isolation

Each sample was diluted as needed, then the last 3 dilutions were poured into 3 petri dishes containing NA by using spread plate method. The dishes containing the sample culture were incubated at 37 °C for 24 h. The single colony was taken and transferred into slanted NA as the host bacterial culture stock^31^.

### Lytic activity test

Into several test tubes containing 7 mL NB, 100 µL of 20-h-old host bacteria were added. A total of 100 µL 0.3 M CaCl_2_ and 100 µL phage were added to the test tube containing the host bacterial culture. The test tubes were incubated at 37 °C and observed every 30 min until significant difference was found in the level of turbidity between controls (which were not infected by bacteriophages) and the test tubes containing host bacteria infected by bacteriophages. The infection time was defined when phage infected the host bacterial cultures after it was cultivated at a particular time. The infection time was done by infecting the phage to the host bacterial cultures (*B. subtilis*) that has been incubated for 0, 30, 60, 90 and 120 min at 37 °C, 75 rpm. Positive test media (the media became clear) were filtered by using a sterile filter to obtain phage lysates (filtrate containing bacteriophages), which were then used for the production of phages^32,33^.

### Bacteriophage production

2.5% of the 20-h-old host bacterial culture was added to the 10 ml TSB medium. 2.5% phage lysate and 0.3 M CaCl_2_, as much as 2.5%, were added to increase phage adsorption to the host bacteria. The phage lysate and host bacterial culture mixture were incubated for 24 h at 37 °C. Then centrifugation was carried out at 8000 rpm for 20 min, and phage supernatant was filtered using a 0.22 mm membrane filtration to yield a bacterial cell-free phage lysate^30^.

### Biofilm reduction test

Biofilms were formulated according to the procedure listed for biofilm confirmation. Once the biofilm was formulated, the SS slide along with the biofilm was transferred to a new medium. Phage lysate was added to the media with a biofilm of 10^7^PFU/mL. The concentration of the biofilm-forming bacteria and final bacteriophages were calculated by using Standard Plate Count (SPC) and double layer agar^34^.

### Biofilm development

Biofilm was generated by the following method^35^: Stainless steel slides (SS slides) were immersed in a 0.2% commercial detergent solution overnight. Afterward, the SS slides were immersed again in a new commercial detergent solution using warm water and agitated for 5 min. SS slides treated with detergent were then washed with distilled water and immersed in new distilled water. The SS slides were then sterilized at 121 oC for 15 min. The sterile SS slide was placed into a 6 cm diameter Petri dish containing 15 ml sterile TSB supplemented by 0.25% of glucose. *B. subtilis* was grown in TSB medium at 37 °C with 75 rpm agitation. 50 µL of *B. subtilis* with OD > 0.24 was put into the TSB medium and added with 20 µL of 0.9% NaCl solution. They were then incubated at 37 °C for 72 h with changing media every 12 h.

## Results and discussion

### Host and Bacteriophage isolation

**Figure 1:**
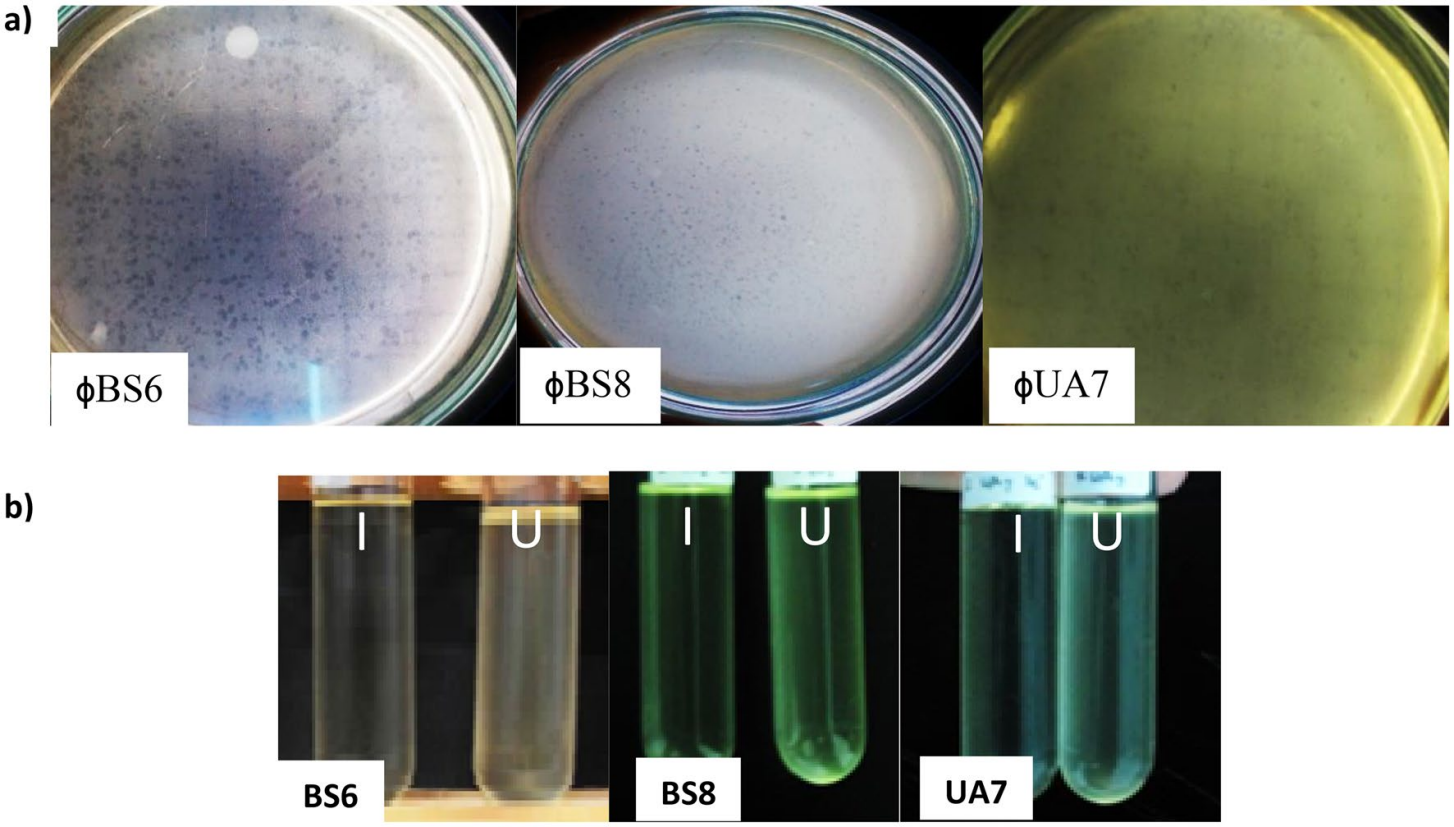
Plaque of phage ϕBS6, ϕBS8 and ϕUA7 on the lawn of BS6 isolate, BS8 isolate and UA7 isolate, respectively (**a**). Turbidity comparison of infected (I) and uninfected (U) on BS6 isolate, BS8 isolate and UA7 isolate, respectively (**b**). The infected tube has the host bacteria lysed by the bacteriophages, thus the turbidity decreased. The uninfected tube only has the host bacteria grown, thus the turbidity increased.

The use of samples of beef tripe and chicken intestines for phage isolation in this work is due to that these samples can be the habitat for *Bacillus subtilis*. Ten isolates of host bacteria from each sample were observed and taken as stock culture for further phage isolation. From 10 isolates of chicken intestines and 10 isolates of beef tripe, three isolates with plaque formation—namely BS6 and BS8 from beef tripe; UA7 from chicken intestines—were observed. Identification by Gram staining confirmed that BS6 is Gram positive, while BS8 and UA7 are Gram negative. The isolation process of phages using BS6, BS8 and UA7 as host strains resulted in the formation of plaque on the agar (Fig. 1a). The plaque formation indicate that bacteriophage is present in the samples. However, phage is specific for some bacteria, thus only several host bacteria isolated from the samples can be infected and shown the plaque formation. Plaque obtained from this isolation show the clear plaques indicated that isolated phage should be lytic phage not temperate one. To prove the lytic activity and the virulence of the bacteriophage, lytic activity test (turbidity test) was performed. By comparing the turbidity of infected and uninfected host bacteria, lytic activity of phage could be observed (Fig. 1b).

From turbidity test, it shown that the infected host bacteria had a decrease in turbidity with significant difference from the uninfected host bacteria. It indicated that the isolated phage had a high lytic activity and potential for use in the application to control the growth of host bacteria. The turbidity level of bacterial cells decreased along with the increase in the number of the infected bacterial cells^32^. It happened because the lysis-causing enzymes produced by the virus would be mixed with bacterial cells and absorbed onto the cell surface breaking the bonds between the bacterial cell walls. The isolation successfully obtained 3 phages that had a high lytic activity against host bacteria, namely ϕBS6, ϕBS8, and ϕUA7. From the results of the lysis of the infected host bacteria, filtration (0.22 μm) and purification was performed to obtain a high concentration of phages.

### Bacteriophage production

**Figure 2:**
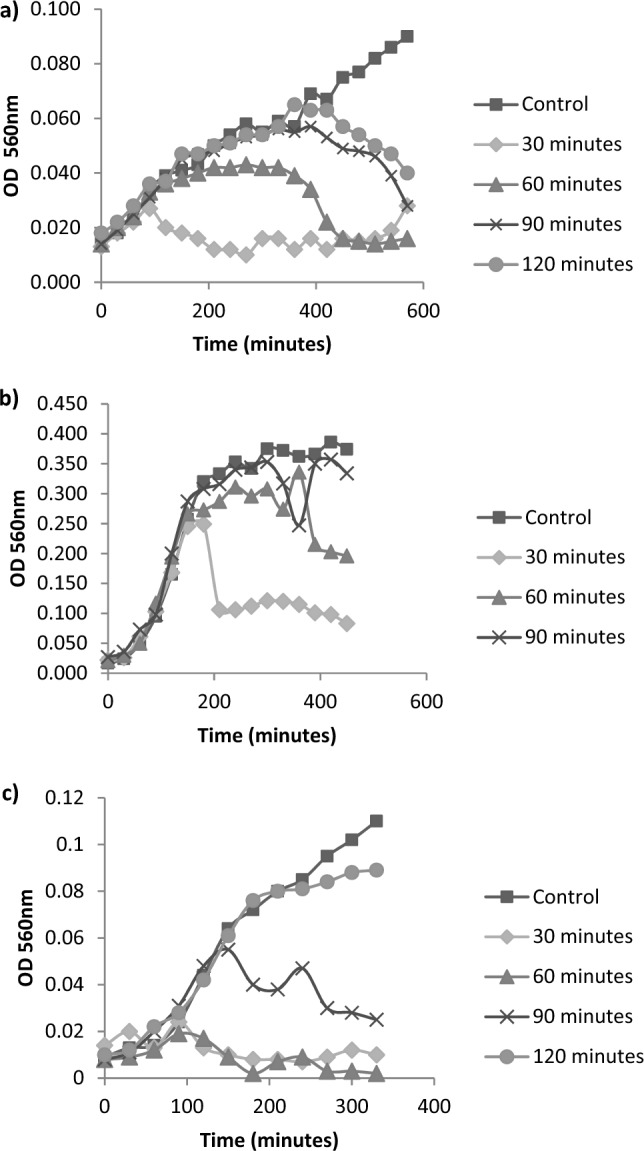
The effect of time infection to the lysing time of bacteriophage in host bacteria (**a**) BS6, (**b**) BS8, and (**c**) UA7.

Phage production was carried out to obtain a high phage concentration. The number of phages collected was influenced by the time of phage infection towards host cells. Figure 2 shows the effect of different phage infection times on the growth of host cell (BS6, BS8 and UA7).

The exact infection time for each bacteriophage was decided based on the growth profile of the host bacteria. The inoculation period of host bacteria that gained an increase in OD and then a significant decrease was used as the right time for the infection process of the host bacteria to increase the concentration of bacteriophages. It was stated that upon the lytic cycle, there found a period where the host cell is still able to live to replicate the bacteriophage after the adsorption is made by the bacteriophage (the period can be depicted by the increase of the host cell)^36^. Furthermore, this period will be followed by a period in which the bacteriophage, which is ready to release, will cause the lysis of the host bacteria, and the new bacteriophage is ready to infect other host cells around (the period is indicated by the decrease in the growth of host cells). Figure 2 shows that the appropriate infection time for each host cell was 90 min for BS6 and UA7 and 30 min for BS8. The timing of phage infection to host bacteria is based on the high lytic ability of phages to produce high concentrations of phage titers. The available host bacterial concentration influences the high titer concentration. The higher the number of host cells available for phage infection, the higher the concentration of phages released during the lytic process, resulting in a high titer of phages. In the case of BS6 and UA7, the infection time was chosen to be 90 min because it can provide a high number of host cells but still allows phages to effectively lyse host cells so that the phage released during the lytic process is relatively high in concentration. The same thing happened in BS8 but at 30 min infection time. After the infection stage, the number of bacteriophages was calculated. The phage propagation process that had been conducted with the available infection time generated the number of phages as shown in Table 1.

**Table 1:** Total bacteriophages of beef tripe and chicken intestine.

Bacteriophage	Total
ϕBS6	1.12 × 10^7^ PFU mL^−1^
ϕBS8	3.00 × 10^4^ PFU mL^−1^
ϕUA7	3.00 × 10^4^ PFU mL^−1^

ϕ = phage symbol, ϕBS = phage isolated from beef tripe, ϕUA = phage isolated from chicken intestine.

### The inhibition spectrum of phage against biofilm-forming bacteria

Several biofilm-forming target bacteria were used to determine the inhibition spectrum of phage isolated (ϕBS6, ϕBS8, dan ϕUA7). The target bacteria were *Pseudomonas fluorescence, Bacillus subtilis, Escherichia coli*, *Listeria monocytogenes*, *Staphylococcus aureus,* and *Salmonella typhi*. The phage inhibition against target bacteria is presented in Table 2.

**Table 2:** Growth inhibition of target bacteria by phages.

Biofilm-forming bacteria	Bacteriophage		
	ϕBS6	ϕBS8	ϕUA7
*Pseudomonas fluorescense*	−	−	−
*Bacillus subtilis*	+	+	+
*Escherichia coli*	−	−	−
*Listeria monocytogenes*	−	−	−
*Staphylococcus aureus*	−	−	−
*Salmonella typhi*	−	−	−

−: no growth inhibition occurs, + : growth inhibition occurs.

**Figure 3:**
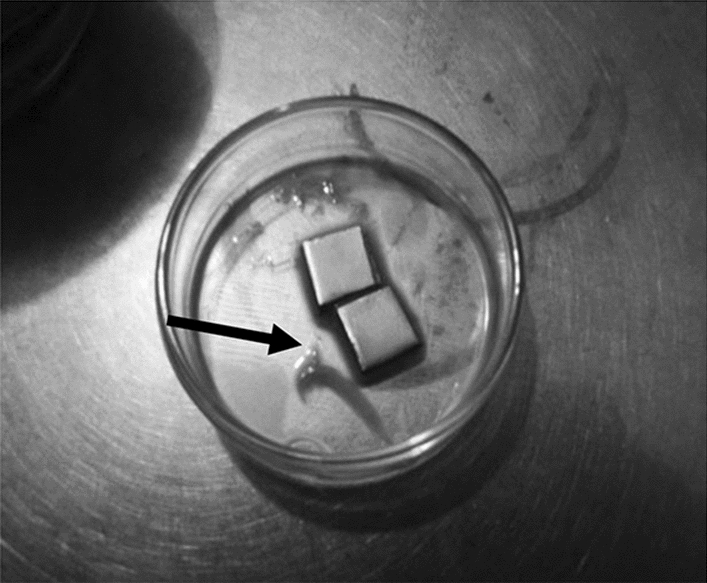
*Bacillus subtilis* biofilm formed under static condition with media renewal every 12 h. $\to$ arrow indicating biofilm formation.

The results indicated that only *B. subtilis* could be inhibited by phages, so *B. subtilis* was chosen as the target bacterium for biofilm formation in the next stage. *B. subtilis* is known as one of the most studied Gram-positive, non-pathogenic, biofilm-producing bacteria in the laboratory due to its ease of morphological and genetical diversification^37^. It was assumed that the infection of *B subtilis* by phages is due to the suitability of phages receptor to *B. subtilis*. Bacteriophage has several receptors for its attachment and it depends as well at the host cell wall component^38^. The inhibitory range of phages is known to be related to the receptors they possess. When a phage has one type of receptor for the host cell, it has a limited inhibitory ability. However, when the phage has more than one receptor, the range of inhibition against the host cell will be broader. This isolated phage may have more than one type of receptor so that it can inhibit both Gram-positive and Gram-negative. Phages are typically highly specific, often being restricted to particular strains within a single bacterial species, however some bacteriophages have a relatively broad host range, infecting multiple species within a genus, and can even infect members of other genera closely related to their usual host. There are big differences in Gram-positive and Gram-negative host receptors, thus narrowing bacteriophage spectrum. From the three host bacteria, BS6 was the only Gram-positive bacteria while the other two (BS8 and UA7) were Gram-negative bacteria. It could be understood that ϕBS6 gave the best inhibition on *B. subtilis* since both *B. subtilis* and BS6 were Gram-positive bacteria, however ϕBS6 could not infect other Gram-positive bacteria such as Listeria and *S. aureus*. Furthermore, ϕBS8 and ϕUA7 could not infect *P. fluorescens, E. coli* and Salmonella even though all of them were Gram-negative bacteria. Based on this fact, it can be assumed that non-infection of the host cell by phages is due to the influence of infection time and duration of phage infection rather than receptor compatibility. Another assumption is that the inability of ϕBS8 and ϕUA7 to infect Gram-negative bacteria are the attachment type. There are two types of phages attachment in bacteria surface—reversible and irreversible attachment. In the reversible attachment, there is a possibility that the phage could be dissociated from the bacteria while the opposite happens in irreversible attachment^39^. It is expected that the two phages (ϕBS8 and ϕUA7) bind reversibly onto the Gram-negative bacteria (*P. fluorescens* and *E. coli* and *Salmonella*) which resulted in improper bacteria inhibition. This assumption could be used as fundamental background for further study of ϕBS8 and ϕUA7 infection behavior. As new host bacteria, *B. subtilis* was reconfirmed its ability to form biofilms (Fig. 3).

**Figure 4:**
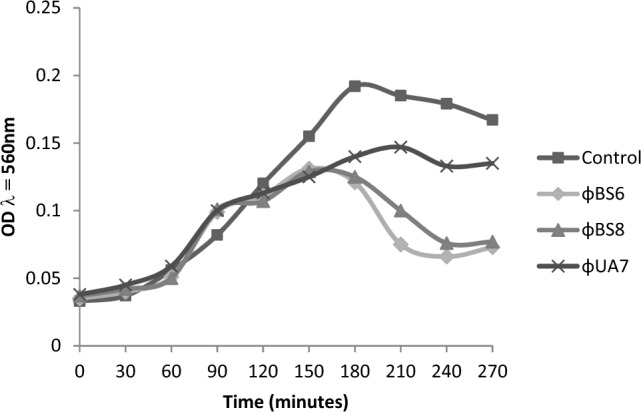
Phage inhibition against *Bacillus subtilis.*

To find out the amount of inhibition per phage against *B. subtilis*, a lytic activity test was conducted against *B. subtilis,* as presented in Fig. 4.

**Figure 5:**
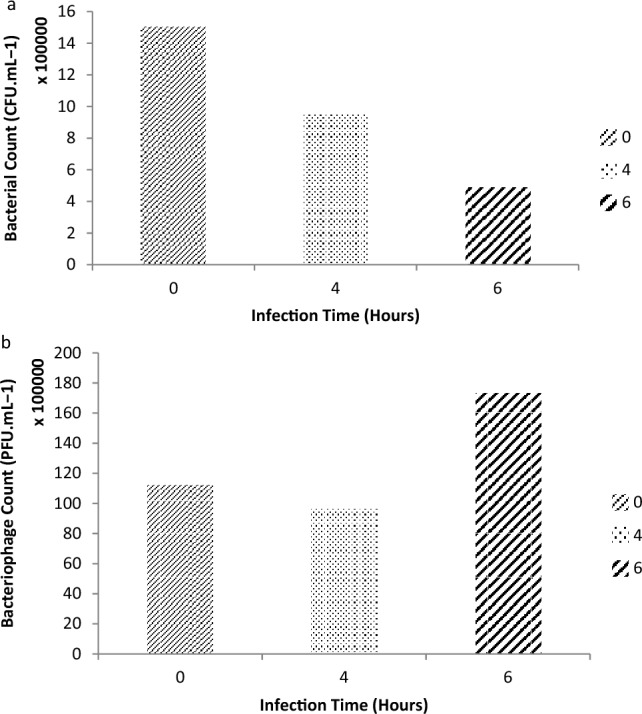
*Bacillus subtilis* biofilm infection using ϕBS6—bacterial count (**a**), phage count **(b**). It was observed that bacterial count was decreased while phage count was increased, indicated that ϕBS6 could inhibit *B. subtilis* biofilm.

The data indicated that ϕBS6 dan ϕBS8 could decrease the growth of *B. subtilis* where ϕBS6 could perform greater inhibition than ϕBS8. Afterwards, the bacteriophage was applied by infecting *B. subtilis* by using ϕBS6 with two different infection times (4 and 6 h). The infection process showed that the total *B. subtilis* bacteria in the biofilm decreased after being infected with ϕBS6 (Fig. 5).

Phage infection in *B. subtilis* indicated that the total number of *B. subtilis* bacteria decreased after being infected with ϕBS6. The control biofilm had a total of 1.50 × 10^6^ CFU mL^−1^ bacteria, while the biofilms infected with ϕBS6 for 4 h and 6 h had a total bacterial of 9.50 × 10^5^ CFU mL^−1^ dan 4.90 × 10^5^ CFU mL^−1^, respectively. The total decrease in *B. subtilis* bacteria obtained was 0.5 log cycle. It is consistent with the study of^40^ that phage ϕ29 begins to have a significant effect on total *B. subtilis* after 48 min of infection. The total ϕBS6 showed that during infection, there happened a decrease in the number of ϕBS6 followed by an increase of 1 log cycle. The initial amount of ϕBS6 added was 1.12 × 10^7^ PFU mL^−1^ while upon biofilm infected for 4 h and 6 h, the total bacteriophages obtained were 9.70 × 10^5^ PFU mL^−1^ dan 1.73 × 10^7^ PFU mL^−1^, respectively (Fig. 5). The decrease indicated that ϕBS6 went into the latent phase, in which the bacteriophage made the adsorption process and inserted its genetic material into the bacteria. It was stated that the latent phase refers to the phase where individual cells are infected, but they do not infect other cells. It implies that during the latent phase, the bacteriophage DNA has entered the bacterial cell and lysis has not occurred^36^. After the latent phase, the bacteriophage will enter the lytic phase, in which the bacteriophage is assembled and ready to be removed from the bacterial cell. In this phase, the number of bacteriophages increases as shown in Fig. 5. These data confirm that ϕBS6 might be a promising candidate for inhibiting the biofilm formation of *B. subtilis*. However, a further study still needs to be performed to identify bacterial isolates susceptible to the tested phages and test the anti-biofilm activity of the mixture of bacteriophages against *B. subtilis* and other target pathogens. Furthermore, an investigation of the potential of these phages in lysing other potential pathogens than *B. subtilis* is necessary to be carried out so that these findings will significantly impact phage treatment for reducing cases of foodborne illness along with antibiotics.

## Conclusions

The isolation process of a bacteriophage using samples of beef tripe and chicken intestine obtained three bacteriophage isolates with lytic properties derived from two isolates of beef tripe (ϕBS6 and ϕBS8) and one isolate of the chicken intestine (ϕUA7). ϕBS6 showed the best infection with the highest decrease in the growth of *Bacillus subtilis*, which was 0.5 log cycle. This study confirms that isolated phages have promising potential as biocontrol agents for biofilm-forming  bacteria.

## Data Availability

All data generated or analyzed during this study are included in this published article.
